# Correlation between plasma renalase level and coronary artery disease

**DOI:** 10.12669/pjms.305.5286

**Published:** 2014

**Authors:** Benhong He, Jianjun Hao, Weiwei Sheng, Yuancai Xiang, Jiemei Zhang, Hao Zhu, Jingcheng Tian, Xu Zhu, Yunxia Feng

**Affiliations:** 1 Benhong He, Department of Cardiovascular Medicine, Lichuan Hospital of Traditional Chinese Medicine, Hubei University of Chinese Medicine, Lichuan 445418, China.; 2 Jianjun Hao, Department of Cardiovascular Medicine, Wuhan No. 1 Hospital, Huazhong University of Science and Technology, Wuhan 430022, China.; 3 Weiwei Sheng, Department of Cardiovascular Medicine, The Central Hospital of EnShi Prefecture, Enshi Clinical College of Wuhan University, Enshi, Hubei 445000, China.; 4 Yuancai Xiang, Department of Cardiovascular Medicine, Lichuan Hospital of Traditional Chinese Medicine, Hubei University of Chinese Medicine, Lichuan 445418, China.; 5 Jiemei Zhang, Department of Cardiovascular Medicine, Wuhan No. 1 Hospital, Huazhong University of Science and Technology, Wuhan 430022, China.; 6 Hao Zhu, Department of Cardiovascular Medicine, Lichuan Hospital of Traditional Chinese Medicine, Hubei University of Chinese Medicine, Lichuan 445418, China.; 7 Jingcheng Tian, Department of Cardiovascular Medicine, Lichuan Hospital of Traditional Chinese Medicine, Hubei University of Chinese Medicine, Lichuan 445418, China.; 8 Xu Zhu, Department of Cardiovascular Medicine, Wuhan No. 1 Hospital, Huazhong University of Science and Technology, Wuhan 430022, China.; 9 Yunxia Feng, Department of Cardiovascular Medicine, Wuhan No. 1 Hospital, Huazhong University of Science and Technology, Wuhan 430022, China.

**Keywords:** Coronary artery disease, Coronary artery stenosis, Renalase, Syntax score

## Abstract

***Objective:*** To explore the correlation between the plasma renalase level of coronary artery disease (CAD) patients and the degree of coronary artery stenosis.

***Methods:*** A total of 180 patients who received coronary angiography in our hospitals from August 2013 to October 2013 were selected as the CAD group, of which 164 were finally diagnosed as CAD. Another 140 healthy subjects were selected as the control group. The plasma renalase levels of the two groups were detected by ELISA to analyze CA-induced changes and to clarify the correlations with the number of branches with coronary artery stenosis and Syntax scores.

***Results:*** The plasma renalase level of the CAD group was significantly lower than that of the control group (P<0.05). The plasma renalase levels of the multi-branch and two-branch stenosis subgroups were significantly lower than that of the subgroup with normal coronary angiography outcomes (P<0.05), while the levels of the single-branch stenosis and normal subgroups were similar (P>0.05). Besides, the plasma renalase level of the low-risk subgroup was significantly higher than those of the medium-risk and high-risk subgroups (P<0.05), and the level of the medium-risk subgroup was significantly higher than that of the high-risk subgroup (P<0.05). Multivariate Logistic regression analysis showed that renalase level was the risk factor of CAD (OR=1.12, 95%CI: 1.03-3.34).

***Conclusion:*** Plasma renalase level was correlated with CAD, the changes of which may reflect the degree of coronary artery stenosis. Therefore, plasma renalase level can be used to indicate the progression of CAD.

## INTRODUCTION

Kidney diseases are closely associated with cardiovascular diseases, and the patients with the former are often prone to the latter.^1^^,^^2^ However, the interaction between them remains unclear, which has been attributed to sympathetic excitement.^3^^,^^4^ Besides expelling waste and excess water as well as maintaining water-electrolyte balance, the kidney also secretes hormones (e.g. renin and erythropoietin) that regulate blood pressure and formation of erythrocytes after entering blood. Therefore, the heart and the kidney regulate human organs simultaneously.^5^^,^^6^

 Nowadays, renal dysfunction has become a high-risk factor for coronary artery disease (CAD). Based on the Mammalian Gene Collection, Xu et al.^7^ cloned an enzyme, renalase, which was highly expressed in human kidney. Renalase, which is closely related with myocardial ischemic changes, carotid atherosclerosis and coronary stenosis, can degrade catecholamines in blood. Considering that clinical studies concerning the correlation between plasma renalase level and CAD remain limited, this study was performed to explore the relationship between such level and coronary artery stenosis and to postulate the possible mechanism.

## METHODS

***Subjects: ***A total of 180 CAD patients who received coronary angiography (CAG) from August 2013 to October 2013 were selected in this study. This study has been approved by the ethics committee of our hospitals. ***Inclusion criteria***: (1) First visit to our hospitals with the main complaints of chest pain and discomfort that required CAG; (2) confirmed CAD that needed further intervention to evaluate the position and degree of coronary artery stenosis; (3) with the need of evaluations on treatment outcomes as well as progression and prognosis of coronary atherosclerosis; (4) with signed written consent. ***Exclusion criteria:*** The patients with cardiomyopathy, heart failure, severe acute and chronic infections, cancer, autoimmune disease, and severe liver and kidney dysfunctions. By using CAG, 164 patients were diagnosed as CAD and the other 16 were not. The average age of CAD patients was (66.36 ± 12.98) years old. There were 110 males (67.1%) and 54 females (32.9%). Another 140 healthy subjects who received physical examinations in our hospitals were selected. The average age was (56.88 ± 18.32) years . There were 70 males and 70 females. They were diagnosed normal by electrocardiogram, chest X-ray and cardiac enzyme examinations, with liver, kidney, cardiovascular and hematological system diseases excluded.

***Diagnosis standards:***

(1) CAD: CAG disclosed that the lumen diameter of ≥1 branch with coronary artery stenosis was ≥50% lower than that of normal ones.

(2) Type 2 diabetic mellitus (T2DM): T2DM or history of T2DM was confirmed according to the Diagnostic Criteria for Diabetes issued by WHO in 1999.

(3) History of smoking: The patients had been smoking for more than one year, ≥1 cigarette per day.

(4) Dyslipidemia: High total cholesterol (TC), triglyceride (TG), high density lipoprotein-cholesterol (LDL-c) and low high-density lipoprotein-cholesterol (HDL-c) levels were determined according to Chinese Guidelines on Prevention and Treatment of Dyslipidemia in Adults (2007).^8^

***Methods:***

***CAG method and evaluation standard for CAD degree:***

(1) CAG method: CAG was selectively performed and the results were evaluated by cardiology physicians with Judkins method.

(2) Number of branches with CAD: CAG disclosed the numbers of CAD-involved branches with the stenosis diameter ≥50% and main branches of coronary artery, mainly including left main artery (LM), left anterior descending artery (LAD), left circumflex artery (LCX) and right coronary artery (RCA). When LM was involved, involved LAD and LCX should also be taken into consideration.

(3) Syntax scoring method^9^: SYNTAX Score Calculator Version 2.02 was downloaded from the official website to evaluate the CAD outcomes of selected cases.

***Grouping methods***

(1) All cases were divided into a CAD group diagnosed by CAG and a normal control group.

(2) The CAG group was further divided into a normal subgroup without CAG abnormality, a subgroup with one branch of stenosis, a subgroup with two branches of stenosis, and a subgroup with multiple branches of stenosis.

(3) The CAG group was further divided into a low-risk subgroup (1~22), a medium-risk subgroup (23~32) and a high-risk subgroup (≥33) according to the Syntax scores.

***Determination of plasma renalase levels: ***Plasma renalase levels were measured by ELISA with human renalase (RNLS/ MAO-C) kit (Shanghai Boyao Biotechnology Co., Ltd.) according to the manufacturer's instructions.

***Examination of clinical and biochemical indices: ***After at least 12 hours of fasting, the patients were subjected to cubital venous blood sampling (approximately 5 ml) in the morning, and serum was separated after anticoagulant treatment to measure the levels of serum TC, TG, LDL-c, HDL-c, uric acid, urea nitrogen and creatinine (UniCel DxC 800 Synchron Clinical Systems, Beckman).

***Statistical analysis: ***All data were analyzed by SPSS 13.0. The numerical data were expressed as (  ± s). Inter-group comparisons were performed by t test and Chi-square test. The qualitative data were compared by Chi-square test. The correlation between plasma renalase level and degree of CAD was analyzed by Pearson's correlation test. Multivariate Logistic regression analysis was used to analyze CAD-related risk factors. P<0.05 was considered statistically significant.

## RESULTS

***Clinical data of CAD group and normal control group: ***Compared with the normal control group, the CAD group comprising more males were older and were more prone to T2DM, with significantly longer history of smoking as well as more carotid atherosclerotic plaques and changes in the ST segment (P<0.05 or P<0.01). However, the renalase level was significantly lower (P<0.05). Meanwhile, the levels of blood TC, TG, HDL-c, LDL-c, creatinine, urea nitrogen and uric acid in the two groups were similar (P>0.05) (Table-I and Table-II).

***Correlation between number of branches with CAD and plasma renalase level: ***Variance test showed that the inter-group F value was 3.08. The plasma renalase levels of the subgroups with multiple-branch and two-branch stenosis were significantly lower than that of the normal subgroup (P<0.05), but the subgroup with single-branch stenosis and the normal group had similar results (P>0.05) (Table-III).

***Correlation between Syntax score and plasma renalase level: ***Variance test showed that the inter-group F value was 3.45. The plasma renalase level of the low-risk subgroup was significantly lower than those of the medium-risk and high-risk subgroups (P<0.05), and the level of the high-risk subgroup was significantly lower than that of the medium-risk subgroup (P<0.05) (Table-IV).

***Risk factors of CAD: ***Logistic regression analysis showed that renalase level, male, age, T2DM and history of smoking were the risk factors for CAD (Table-V).

## DISCUSSION

The degree of CAD is associated with renal functions.^10^ The patients with mild chronic renal dysfunction are more prone to CAD even without conventional risk factors.^11^ Therefore, renal function has long been considered as a high risk factor for CAD, but the detailed action mechanism remains mystery. Probably, the fluctuation of renalase level may be responsible.

As a monoamine oxidase relying on flavin adenine dinucleotide, renalase is the only enzyme which has been verified hitherto to secrete catecholamines that enter the blood and metabolic cycle. Being related with cardiovascular events, renalase exerts regulatory effects on sympathetic activity. With aggravated renal function injuries, the levels of plasma renalase gradually drop.^12^ Li et al.^13^ found that the patients with chronic kidney disease, especially those in the end stage, underwent evident decreases in the renalase level.

Renalase can protect ischemic myocardium and reduce the area of myocardial infarction. Desir et al. reported that the mice with knockout renalase gene were more vulnerable to myocardial ischemia than the control mice did.^14^ Moreover, the incidence of myocardial infarction could be reduced by 54% after injection of renalase. In this study, the CAD patients had lower plasma renalase levels than the normal subjects did, but the levels of serum creatinine, urea nitrogen and uric acid were similar, indicating that renalase had participated in the onset and progression of CAD before obvious weakening of renal function. According to the CAG results, the degree of CAD was quantified by the number of involved branches. Plasma renalase level decreased in the patients with CAD, particularly in those with two branches and more involvement. As an angiography scoring system completely based on the coronary anatomical and pathological characteristics, syntax score has been demonstrated to be able to predict the clinical outcomes of patients with stable multivessel disease. In this study, the plasma renalase levels of 164 CAD patients gradually reduced with increasing Syntax score. Hence, plasma renalase level was related with both the onset and the development of CAD, which dropped with aggravating CAD.

**Table-I:** Clinical data of CAD group and normal control group

***Group***	***N***	***Male***	***T2DM***	***History of smoking***	***Carotid atherosclerotic plaque***	***Change in ST segment***
CAD	164	110/164 (67.1%)	60/164 (36.6%)	70/164 (42.7%)	66/164 (74.4%)	88/164 (69.5%)
Normal control	140	70/140 (50%)	26/140 (18.6%)	28/140 (20%)	116/140 (45.7%)	100/140 (42.9%)
Chi-square value		6.068	6.043	8.894	13.075	10.962
P value		<0.05	<0.05	<0.05	<0.01	<0.01

**Table-II:** Clinical and biochemical indices of CAD group and normal control group

***Group*** ***(Unit)***	***TC*** ***mmol/L***	***TG*** ***mmol/L***	***HDL-c*** ***mmol/L***	***LDL-c*** ***mmol/L***	***Age*** ***years old***	***Creatinine*** ***μmol/L***	***Urea nitrogen*** ***mmol/L***	***Uric acid*** ***μmol/L***	***Renalase*** ***ng/L***
CAD	4.59±1.26	1.66±1.02	1.12±0.56	2.70±0.88	66.36±12.98	81.70±34.08	6.18±3.28	361.22±123.34	112.50±12.87
Normal control	4.25±1.25	1.65±1.66	1.11±0.11	2.42±1.05	56.88±18.32	83.83±25.15	6.02±2.05	349.86±115.23	120.79±14.39
t value	1.453	0.093	0.1	1.59	3.372	-0.37	0.31	0.51	2.34
P value	>0.05	>0.05	>0.05	>0.05	<0.05	>0.05	>0.05	>0.05	<0.05

**Table-III:** Plasma renalase levels of subgroups with different branches of stenosis

***Group***	***N***	***Renalase (ng/L)***
Normal	16	120.87±16.31
Single-branch stenosis	64	116.54±6.44&
Two-branch stenosis	56	111.18±9.63*
Multiple-branch stenosis	44	109.21±11.47*

Compared with the normal subgroup,

* P<0.05,

& *P*>0.05.

**Table-IV:** Plasma renalase levels of subgroups with different Syntax scores

***Group***	***N***	***Renalase***
Low-risk	78	117.23±13.24
Medium-risk	60	113.27±14.72*
High-risk	26	109.24±13.43*#

Compared with the low-risk subgroup,

* P<0.05; compared with the medium-risk subgroup,

# P<0.05.

**Table-V:** Logistic regression analysis of risk factors of CAD.

***Variable***	***β***	***SE***	***Wald*** ***χ***^2^	***OR value***	***95%CI***
Male	1.43	0.42	9.63	2.46	2.10-8.32
Age	0.09	0.02	8.73	1.03	1.01-1.64
T2DM	1.23	0.35	10.87	2.34	1.98-5.72
History of smoking	0.56	0.26	12.53	1.32	1.21-4.53
Renalase level	0.26	0.32	9.21	1.12	1.03-3.34

Logistic regression analysis showed that renalase level was the risk factor for CAD. As a conventional risk factor for CAD, dyslipidemia no longer showed statistically significant differences between the CAD group and the control group, which may be ascribed to the administration of lipid-controlling agents before admission.

Renalase decrease may trigger CAD following the mechanisms below: (1) Such decrease raised the concentrations of plasma catecholamines, which enhanced sympathetic activity and then increased the risks of hypertension, and the latter accelerated CAD by increasing the load of cardiovascular system and by promoting arteriosclerosis. (2) The lack of renalase enhanced the oxidative stress induced by norepinephrine and epinephrine, facilitated cell apoptosis, enlarged the area of myocardial infarction, and rendered the area more prone to fibrosis.^15^^,^^16^ It has previously been reported that microinflammation state and degree of oxidative stress were closely associated with deterioration of renal function, as well as CAD mortality and total mortality. Nevertheless, the correlation between renalase decrease and the systemic inflammatory state and oxidative stress level of patients still needs further studies.

In summary, plasma renalase level not only indicated the injury degree of renal function, but also reflected the progression of CAD. As an index for evaluating CAD in clinical practice, undesirable changes of renalase level may be one of the reasons responsible for chronic renal impairment. However, whether renalase replacement therapy can improve the prognosis of CAD patients complicated with chronic kidney disease still requires in-depth studies. Studies based on larger-sized samples are still ongoing in our group.

## Authors’ contributions:

**BH and JH:** Study design and manuscript preparation.

**WS, YX, JZ, HZ, JT, XZ and YF:** Data collection and analysis.

## Notes

***C*** ***onflicts*** ***of interest******: ***The authors declare that they have no conflicts of interest.
